# P02.124. A pilot randomized controlled trial of yoga for prediabetes

**DOI:** 10.1186/1472-6882-12-S1-P180

**Published:** 2012-06-12

**Authors:** K McDermott

**Affiliations:** 1 Univ of California, San Francisco, Osher Center for Integrative Med, San Francisco, USA

## Purpose

To test the effects of a yoga-based intervention on changes in prediabetes measures.

## Methods

We conducted an 8-week, randomized, waitlist controlled trial of yoga for prediabetes (diagnosed by two measures of fasting glucose between 100-125 mg/dl) in Bangalore, India. Forty-one participants were randomized to yoga (n=21, 1 lost to follow-up) or control (n=20, 2 lost to follow-up). All participants attended an 8-hour session on lifestyle changes. Participants in the yoga group also attended 3 to 6 hour-long yoga classes per week. Yoga classes included didactic training, postures and breathing exercises. We measured changes in prediabetes using a 75 gm oral glucose tolerance test (OGTT) with a 2-hour post-prandial blood draw. Fasting glucose and insulin were used to calculate homeostatic model assessment-insulin resistance (HOMA-IR); we also measured mood and perceived stress.

## Results

There were significant differences in mean change scores (yoga – control) for weight (-2.3 kg, 95%CI[-4.1, -0.4]), waist circumference (-3.8 cm, 95%CI[-7.6,-0.05]), and BMI (-0.79 95%CI[-1.6, -0.003]). There were also significant improvements in total cholesterol, systolic and diastolic blood pressure, and psychological assessments in both groups during the study. The yoga group had greater improvements than the control group in 2-hour glucose levels on the OGTT (11.4 vs -1.5) and HOMA-IR (-1.1 vs 0.27) but these were not statistically significant. Fasting glucose did not improve in the yoga group compared with the control group.

## Conclusion

The yoga group had significant improvements in weight and waist circumference. Both groups improved in cholesterol and blood pressure, possibly due to the lifestyle session. There were trends toward improved glucose control on the OGTT and decreased insulin resistance, though fasting glucose did not show evidence of improvement. The data support promise for yoga-based interventions in prediabetes, and suggest that further optimization of intervention content and duration may be useful.

